# ReDisulphID: A discovery platform for thiol redox sensors identifies a druggable site regulating p53 activation

**DOI:** 10.1016/j.redox.2026.104196

**Published:** 2026-04-29

**Authors:** Pierre Coleman, Anna Laddach, Rhys Anderson, Xiaoping Yang, Ravi Kumar, Ajay Shah, Franca Fraternali, Joseph R. Burgoyne

**Affiliations:** a School of Cardiovascular and Metabolic Medicine & Sciences, King's College London, The British Heart Foundation Centre of Excellence, The Rayne Institute, St Thomas' Hospital, London, SE1 7EH, UK; b Nervous System Development and Homeostasis Laboratory, the Francis Crick Institute, 1 Midland Road, London, NW1 1AT, UK; c Proteomics Facility, Centre of Excellence for Mass Spectrometry, King's College London, The James Black Centre, Denmark Hill Campus, London, SE5 9NU, UK; d School of Cardiovascular and Metabolic Medicine & Sciences, King's College London, The British Heart Foundation Centre of Excellence, James Black Centre, 125 Coldharbour Lane, London, SE5 9NU, UK; e Institute of Structural and Molecular Biology, University College London, London, WC1E 6BT, UK; f Research Department of Structural and Molecular Biology, Division of Biosciences, University College London, London, WC1E 6BT, UK; g Department of Biological Sciences, Birkbeck, University of London, London, WC1E 7HX, UK

**Keywords:** Redox sensor, Thiol, Disulphide, PEPD, p53

## Abstract

Thiol redox sensors in proteins are emerging as key therapeutic targets, as they govern fundamental signalling pathways and provide crucial sites for covalent drug development. A key mediator of their function is the presence of redox-active disulphides, which act as molecular switches due to their ability to induce reversible protein conformational changes. However, despite their importance in cellular regulation and therapeutic relevance, only a limited number of redox-active disulphides have been identified to date. To address this, we developed ReDisulphID, a structural bioinformatics platform that systematically identifies druggable redox-active disulphides. Using this platform, we discovered novel druggable redox sensors in MLYCD, TFIIB, and PEPD. Functional analysis of PEPD revealed that its redox sensor activates the tumour suppressor p53. Furthermore, we identified a compound that activates p53 through direct thiol modification of the sensor in PEPD, demonstrating how ReDisulphID can advance the discovery of protein redox sensors and support thiol-targeted drug development.

## Highlights

• Introduces a novel resource for identifying druggable redox sensors.
• Reveals novel redox sensors in MLYCD, TFIIB, and PEPD.
• Discovers a druggable, redox-dependent mechanism for activation of p53.

## Graphical abstract

**Figure:**
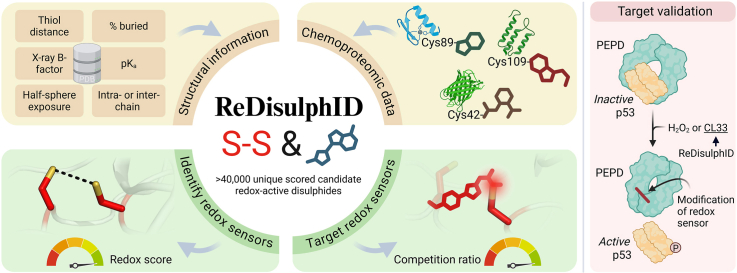

## 1 Introduction

Thiol redox sensors are critical mediators of redox signalling, enabling cells to detect oxidants and convert these signals into specific biological responses. Many such sensors rely on the formation of redox-active disulphide bonds that act as functional switches by promoting specific structural conformations. These conformational changes can alter protein activity, interactions, or subcellular localisation, to modulate downstream signalling pathways [1]. Such redox-driven disulphide mechanisms are increasingly recognised as central to diverse physiological and pathological processes, including blood pressure regulation, angiogenesis, autophagy, and the DNA damage response [[2], [3], [4], [5]].

Reactive cysteine thiols, central to redox signalling, represent promising therapeutic targets through their ability to react selectively with small-molecule electrophiles. Despite previous concerns regarding the safety of covalent drugs, it is now well recognised that such compounds often offer enhanced potency, selectivity, and duration of action compared to non-covalent drugs [6,7]. Furthermore, conventional drug development can only target an estimated 10-15% of the human proteome [8], prompting an increased interest in the development of covalent drugs that can address previously undruggable disease targets [9]. This is being accelerated by the development of chemoproteomic approaches to comprehensively map thiol ligandability, which can be aligned onto known functional sites to facilitate the discovery of lead compounds [10].

Several established therapeutics act via cysteine modification, for instance, gastroesophageal reflux drug omeprazole forms a mixed disulphide with cysteines on the luminal side of the H^+^/K^+^-ATPase pump, primarily C813, to block proton transport and so gastric acid secretion [11]. Cysteine-targeting anti-cancer drugs include afatinib, which targets a cysteine on ErbB family receptors [12], and ibrutinib, which targets C481 of Bruton's tyrosine kinase [13]. Redox-regulated disulphide bonds also offer therapeutic potential in cardiovascular disease. The covalent antiplatelet drug clopidogrel was recently shown to be better than aspirin for indefinite treatment of coronary artery disease [14]. Clopidogrel modifies C97 in the platelet receptor P2Y_12_ to prevent intermolecular disulphide formation that promotes platelet aggregation [15]. Conversely, in the PKG1α homodimer, the intermolecular disulphide between C42 residues is a well-characterised beneficial redox switch that can be induced by dietary polyphenols to lower blood pressure [16,17], highlighting the diverse druggable functions of redox-regulated disulphides.

Despite their functional importance and therapeutic potential, relatively few reversible redox-regulated disulphide bonds have been identified. To address this gap, we developed a high-throughput computational screen that predicts drug-targetable redox-dependent disulphide bonds based on structural features including thiol separation and local environment. This dataset was used to generate ReDisulphID, a platform for discovering redox sensors. This resource identified thousands of putative novel redox sensors, and validation of several top candidates with druggable sites revealed new redox sensors in MLYCD, TFIIB, and PEPD. Further characterisation of the redox sensor in PEPD showed that it can activate the tumour suppressor p53. Using this resource, a small molecule, CL33, was identified that activates p53 via covalent modification of cysteine 58 (C58) in PEPD. These findings establish ReDisulphID as a novel platform for systematically identifying redox-regulated proteins and accelerating the discovery of new thiol-targeted therapeutics.

## 2 Materials and methods

### 2.1 Development of the ReDisulphID platform

All structures in the RCSB protein data bank (PDB) were analysed. The PDB is an open access repository of biological macromolecules determined by X-ray crystallography, NMR, or cryo-EM [18]. Structures were screened for cysteines with thiols <10 Å apart using Python with the Biopython package [19]. Additional information was collected from the UniProt protein database [20], including accession codes and peptide sequences. Flanking regions of cysteines were compared between structures and UniProt sequences to assign standardised residue numbers to cysteines, based on residue numbers in UniProt. When multiple structures were found for the same cysteine pair, the cysteine pair with the shortest thiol separation was kept. pK_a_ was calculated with prediction engine PROPKA3 [21]. HSE is a count of the number of amino acids in a 12 Å-radius half-sphere around either the side chain or backbone around an amino acid. HSE-up corresponds to the half-sphere in the direction of the residue chain, while HSE-down corresponds to the opposing half-sphere. Ligandability data were obtained from Boatner et al. [22], which set out ligandability criteria and gathered quantitative chemoproteomics data from 6 sources [[23], [24], [25], [26], [27], [28]]. The ReDisulphID frontend was rendered statically in HTML using Python. Structures are displayed using 3Dmol.js [29].

Positive candidates of reversible disulphide formation were selected on stringent criteria where site-specific cysteine mutagenesis prevented disulphide-dependent higher MW complex formation detected by immunoblot analysis, and that intermolecular disulphide formation occurred within the endogenous cellular protein. For each putative bond we gathered a set of numeric descriptors: inter-cysteine Sγ–Sγ distance, % buried, HSE total, minimum pK_a_, and average B factor. For each descriptor xᵢ, a Spearman correlation with the classification in the training set of known redox-regulated disulphides (rᵢ) was computed. Highly inter-correlated descriptors (|r| ≥ 0.80) were grouped (% buried and HSE total); inside these groups, weights wᵢ were made proportional to the descriptor's variance. The score S for candidate k was calculated by:

$$S_{k}=\sum_{i}w_{i}r_{i}x_{ik}$$

This gives a single number that was then normalized, for readability, to the smallest ($S_{\min}$) and largest ($S_{\max}$) values, giving a final percentage-based Redox Score using equation:

$$Redox\;Score=\frac{S_{k}-S_{\min}}{{S_{\max}-S}_{\min\;}}\times 100$$

### 2.2 Cell culture and treatment

All cells were maintained at 37 °C in a 5% CO_2_ humidified incubator and were passaged every 2-3 days. HEK293T cells (ATCC, CRL-3216) and HT1080 cells (a gift from Professor Brian Stramer) were cultured in Dulbecco's Modified Eagle Medium (Gibco™, 31,966,021) supplemented with 10% foetal bovine serum (FBS) (PAN-Biotech, P40-39500) and 1% penicillin/streptomycin (Gibco™, 15140122). Cells were seeded 24 h before treatment. Administration of chemical reagents was in DMEM unless otherwise stated. Treatments included H_2_O_2_ (Merck, H1009) or the compounds CL54: 2-chloro-N-(2,2-difluoro-1,3-dioxaindan-5-yl)acetamide (Chemspace, CSSB00000183876); CL53: 2-chloro-N-(2-cyanoethyl)-N-(3,4-dimethylphenyl)acetamide (Chemspace, CSSB00000735026); CL51: 2-chloro-1-[2-(thiophen-3-yl)pyrrolidin-1-yl]ethan-1-one (Chemspace, CSSB00000742156); CL33: 2-chloro-N-[4-(4-methoxy-phenyl)-thiazol-2-yl]-acetamide (Santa Cruz, 6202-74-0). Compound concentrations were defined from preliminary dose-finding studies for p53 activation. For CL53 and CL54, the maximal tested concentration (400 μM) was used due to lack of activation.

### 2.3 Immunoblotting

Samples were collected in 50 mM Tris-HCl pH 6.8, 2% w/v SDS, 10% glycerol, 0.0025% w/v bromophenol blue and 100 mM maleimide. To reduce samples these were supplemented with 5% βME and then boiled for 5 min. Samples were loaded onto Mini-PROTEAN® TGX™ Gels (Bio-Rad, 456-1086) before electrophoretic transfer onto PVDF membranes using a Trans-Blot Turbo Transfer System (Bio-Rad). Membranes were blocked with 10% (w/v) milk in phosphate-buffered saline with 0.1% v/v Tween (PBS-T), then incubated with relevant primary antibodies at 1:1000 overnight. Membranes were washed with PBS-T and then incubated with relevant secondary HRP-conjugated antibody for 1 h. After subsequent washing, membranes were incubated in SuperSignal™ West Pico PLUS Chemiluminescent Substrate (Thermo Scientific, 34580) and imaged using iBright™ CL1500 Imaging System (Invitrogen). The following antibodies were used: Mouse IgG-HRP (Cell Signalling, 7076), Rabbit IgG-HRP (Cell Signalling, 7074), FLAG (Merck Life Science, F1804), Myc (Cell Signalling, 5698S), GAPDH (Cell Signalling, 2118S), PEPD (Proteintech, 12218-1-AP), MLYCD (Bio-Techne, NBP1-32797), TFIIB (Santa Cruz, 271736), p53 pSer15 (Cell Signalling, 9284S).

### 2.4 Analysis of recombinant PEPD

Recombinant PEPD (Bio-Techne, NBP2-51987) incubated with or without 10 mM DTT for 1 h and analysed by SDS-PAGE followed by analysis by Coomassie stain (10% acetic acid, 40% methanol, and 0.1% Coomassie Brilliant Blue). For mass spectrometry analysis, recombinant PEPD was reduced by 1 h incubation with 10 mM DTT, then desalted into 100 mM HEPES and incubated with 2 mM CL33 for 2 h at room temperature.

Protein was buffer-exchanged into 8 M urea using 3 kDa MWCO filters (Amicon Ultra, 0.5 ml) and digested on-filter with trypsin (1:100, enzyme:protein) overnight at 37 °C. Digests were acidified (1% acetonitrile, 0.1% TFA), peptides collected by centrifugation, desalted on C18, and dried by SpeedVac prior to LC–MS/MS. Samples were subsequently analysed using an Orbitrap Eclipse Tribrid mass spectrometer (Thermo). MS data were searched against the sequence of recombinant PEPD (1-493) with a N-Terminal His-tag as per Bio-Techne using Proteome Discoverer 2.5, with standard tryptic parameters and dynamic modifications for methionine oxidation and cysteine CL33 adduction. Peptide identifications were filtered at 1% FDR, and CL33-modified peptides were accepted only with ptmRS site probability = 100.

### 2.5 Transfection and plasmid mutagenesis

Constructs were transfected into mammalian cells using Lipofectamine 3000 Reagent (Invitrogen, L3000008) following the manufacturer's instructions. pcDNA3.1-C-(k)DYK-ACAA2 was obtained from GenScript (Ohu08929D). pCMV3-C-Myc-IRAK4, pCMV3-C-Myc-Mark3, and pCMV3-C-FLAG-PEPD were obtained from Sino Biological (HG10735-CM, HG12181-CM, HG14525-CF respectively). PEPD C58A and C158A mutants were generated using Q5® Site-Directed Mutagenesis Kit (NEB, E0554S) with the following primer-pairs: C58A: TCAGCGCTACGCCACCGACACCGG/GTCTCCTCCCCGCCCTGA, C158A: CGGCAGTGTCGCCAGGGAGGCCTC/CTGTCCGTGTTGACGCCA.

### 2.6 Generation of stable cell lines

PEPD was cloned from pCMV3-C-FLAG-PEPD into pLenti CMV Puro DEST backbone (Addgene, gift from Eric Campeau & Paul Kaufman, 17452) using the following primers TGTACAAAAAAGCAGGCTTAATGGCGGCGGCCACCGGA/TTTGTACAAGAAAGCTGGGTTTACTTATCGTCGTCATCCTTGTAATCAGAGCCTCCACCC for PEPD amplification, and primers ACCCAGCTTTCTTGTACAAAG/TAAGCCTGCTTTTTTGTACAAAC for backbone amplification, followed by assembly with NEBuilder® HiFi DNA Assembly Master Mix (E2621S) to make pLenti-PEPD-CMV. The SV40 promoter was amplified from pSLIK-GFP (Addgene, gift from Christian Metallo, 66844) using primers TTGGGAGTTCCGCGTTACATGTGTGTCAGTTAGGGTGTG/TTTGCAAAAGCCTAGGCC, and inserted into pLenti-PEPD-CMV, which was amplified without the CMV promoter using primers GAGGCCTAGGCTTTTGCAAATCGTTTAGTGAACCGTCAG/ATGTAACGCGGAACTCCC, again using NEBuilder® HiFi DNA Assembly Master Mix, to produce WT pLenti-PEPD. In this construct, PEPD also underwent C58A mutation as described earlier.

HT1080 cell lines stably expressing WT or C58A PEPD were generated with lentiviral infection as previously described with few deviations [30]. Briefly, HEK293FT cells were transfected with WT or C58A pLenti-PEPD plus pMDLg/pRRE, pRSV-Rev, and pMD2.G (Addgene, gifts from Didier Trono, respectively 12251, 12253, 12259). Media was changed 24 h post-transfection and lentivirus-containing media harvested after a further 24 h, which was cleared through a 0.45 μm filter. HT1080 cells were suspended and combined with a serial dilution of lentivirus-containing media and supplemented with 8 μg/mL of polybrene (Santa Cruz, sc-134220) before seeding on 12-well plates. Infection media was replaced with normal media after 24 h and then after a further 24 h were selected with 2 μg/mL puromycin (Gibco™, A1113803) for 48 h. Viral titre in HT1080 cells was quantified by crystal violet colony formation assay after a further 7 days. Cell wells at titres with multiplicity of infection <0.1 were expanded as stable heterogeneous lines.

### 2.7 PEPD activity assay

The activity of PEPD was assessed as previously described [31]. Cell lysates with or without PEPD overexpression were collected in 50 mM HEPES, 150 mM NaCl, 1% Triton X-100, and cOmplete Protease Inhibitor Cocktail (Roche, 11697498001), incubated for 30 min at 4 °C, clarified by centrifugation at 12,000 g for 10 min, and adjusted to 500 μg/mL protein. Lysates were treated with or without 200 μM H_2_O_2_ at 37 °C for 15 min. After incubation, a sample of lysate was taken for immunoblot analysis. In addition, 25 μL of the remaining lysate from each sample was supplemented with 50 μL of 0.5 mM Gly-Pro (MCE, HY-W016887) and 25 μL of 100 mM borate buffer (pH 8) with 1 mM MnCl_2_ before incubation for a further 20 min at 37 °C. Samples were heated to 90 °C for 10 min and snap-frozen in liquid nitrogen. For analysis, each sample was supplemented with 100 μL of 1.25 mM NaIO_4_, 100 μL of 125 mM borate buffer, and 100 μL of 0.75 mM 3,4-dihydroxyphenylacetic acid (Fluorochem, F092681), before incubation at 37 °C for 10 min. After incubation, samples were cooled on ice and briefly centrifuged before fluorescence measurement using a plate reader (Thermofisher, Varioskan), with excitation at 345 nm and emission at 450 nm.

### 2.8 Metabolomics

HT1080 cells stably expressing either WT or C58A PEPD underwent metabolite extraction using a methanol/chloroform/water protocol. Briefly, cells were lysed with methanol/water at 1:1 v/v, then supplemented lysate/chloroform at 2:1 v/v. Lysates were agitated for 30 min at 4 °C and centrifuged for 5 min at 16,000 g. The polar upper phase was kept for LC-MS analysis, which was performed using a 1290 Infinity II UHPLC system coupled with a 6546-Q-TOF mass spectrometer (Agilent Technologies).

## 3 Results

### 3.1 Structural characteristics for predicting redox-regulated disulphide formation

**Fig. 1:**
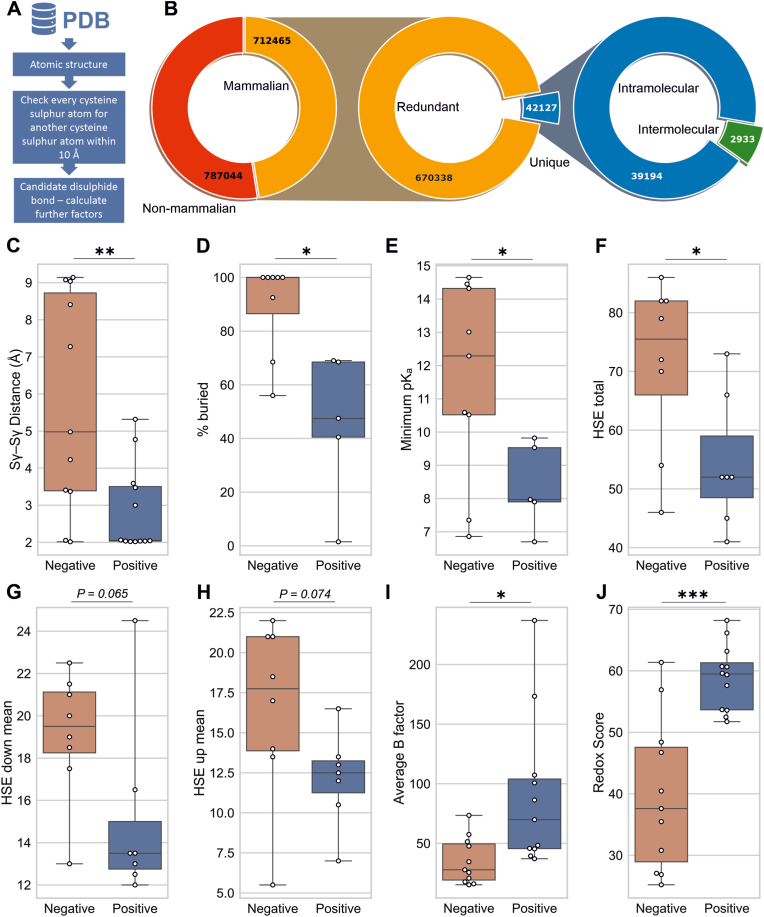
Structural characteristics of redox-regulated disulphides. (**A**) Schematic summarising initial screen. (**B**) Sequence alignment is used to consolidate 712,465 proximal mammalian cysteine pairs to 42,127 unique candidate pairs. Several characteristics were significantly different between known redox-regulated disulphides and negative controls, including (**C**) thiol distance (n = 11, 12), (**D**) % buried (n = 8, 5), (**E**) minimum pK_a_ (n = 9, 5), and (**F**) the number of amino acids within proximity of the cysteines (total HSE, n = 8, 7). Whereas there was a trend for a difference in (**G**) the number of amino acids around the cysteine side chains (HSE up) and (**H**) around the backbone of the cysteines (HSE down). (**I**) Crystal structure B-factor compared between known redox-regulated disulphides and negative controls (n = 11, 11), (**J**) Redox Score, calculated by combining structural features, provides improved separation of known redox-regulated disulphides from negative controls (n = 11, 12). ∗P < 0.05, ∗∗P < 0.01, ∗∗∗P < 0.001 by Welch's two tailed *t*-test.

The PDB was screened for thiol pairs within 10 Å of each other (Fig. 1A). Here 1,499,509 cysteine pairs were detected, which were de-duplicated with UniProt sequence alignment to leave 42,127 non-redundant mammalian pairs. This comprised 39,194 intramolecular cysteine pairs and 2933 that were intermolecular, which were used to compile the ReDisulphID database (Fig. 1B). To predict cysteine pairs likely to undergo redox-dependent disulphide formation, we compiled a library of cysteine pairs known to form redox-dependent disulphides, as well as other cysteine pairs within the same proteins unable to form this modification (Fig. S1A). Additional negative controls were included by confirming that interchain cysteine pairs in IRAK4, MARK3, and ACAA2 do not form intermolecular disulphides (Fig. S1B–D). Structural parameters were then compared between cysteine pairs positive and negative for redox-dependent disulphide formation. From those assessed, thiol distance, minimum pK_a_ and measures of solvent accessibility (% buried and total half-sphere accessibility (HSE)) were significantly different between the two groups (Fig. 1C–H). Thiol distance was still significantly shorter in those positive for redox-dependent disulphide formation when crystallographic disulphide bonds were excluded (Fig. S2). We found that cysteine pairs in known redox-regulated disulphides had higher B-factors than those unable to form this modification (Fig. 1I). Local B-factor is influenced by protein heterogeneity across a crystal [32,33], consistent with redox-regulated disulphides having a mixed population of bound and unbound forms throughout. By combining these structural characteristics, a redox disulphide prediction algorithm was generated that produced a redox score, indicating the likelihood of cysteine pairs in forming redox-dependent disulphides. The redox score provided a stronger indicator of redox sensitivity to disulphide formation compared with using individual parameters alone (Fig. 1J).

### 3.2 ReDisulphID enables discovery of drug-targetable redox-regulated disulphides

**Fig. 2:**
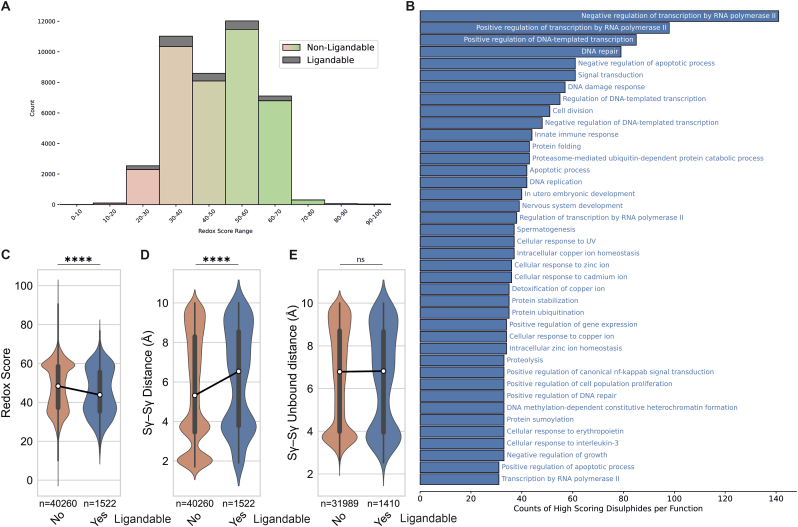
ReDisulphID identifies novel candidate drug-targetable redox-regulated disulphides. (**A**) The number of potential redox-regulated disulphides is shown in relation to the Redox Score and the proportion that have identified ligands. (**B**) Function analysis of proteins with ligandable cysteine pairs that have scores above the lowest-scoring positive candidate of Redox score 53, showing the top 40 functions. (**C**) Redox scores for cysteine pairs identified with or without ligands. Thiol distance for cysteine pairs identified with or without ligands using (**D**), the complete dataset, or (**E**), after removal of those disulphide-bound within the protein structure. (**C-E**) ^ns^P > 0.05, ∗∗∗∗P < 0.0001 by Mann-Whitney U non-parametric test.

All candidate cysteine pairs were compiled into a user-accessible database termed ReDisulphID (Fig. S3). In addition to the redox score, we also mapped chemoproteomically identified sites of ligandability onto cysteine pairs within the ReDisulphID database [22]. Many mapped ligands could modify thiols within a cysteine pair that had a high redox score (Fig. 2A). These high-scoring ligandable cysteine pairs occurred in proteins with a diverse range of functions, particularly in transcription regulators and DNA repair proteins (Fig. 2B). However, there was also a large proportion of ligands that modify cysteines with a low predicted redox score. This was surprising given the factors that would be expected to make a thiol more amenable to adduction by small molecules, such as increased solvent accessibility, make a cysteine pair more redox-active. To investigate this further we compared redox scores between ligandable and non-ligandable candidate cysteine pairs. Here we found that ligandable pairs were less likely to undergo reversible disulphide formation than non-ligandable pairs (Fig. 2C). We rationalized that this may be due to disulphide formation limiting ligand binding. This was consistent with non-ligandable cysteine pairs having shorter Sγ–Sγ separations, with a peak at ∼2 Å that represents cysteine pairs that are disulphide-bound within the structure (Fig. 2D). When disulphide-bound cysteines were excluded from the analysis the relationship between distance and ligandability disappeared (Fig. 2E). These findings suggest that redox-dependent disulphide formation may limit the ability to identify ligands capable of modifying these cysteines.

### 3.3 Novel redox-regulated disulphides identified by ReDisulphID

**Fig. 3:**
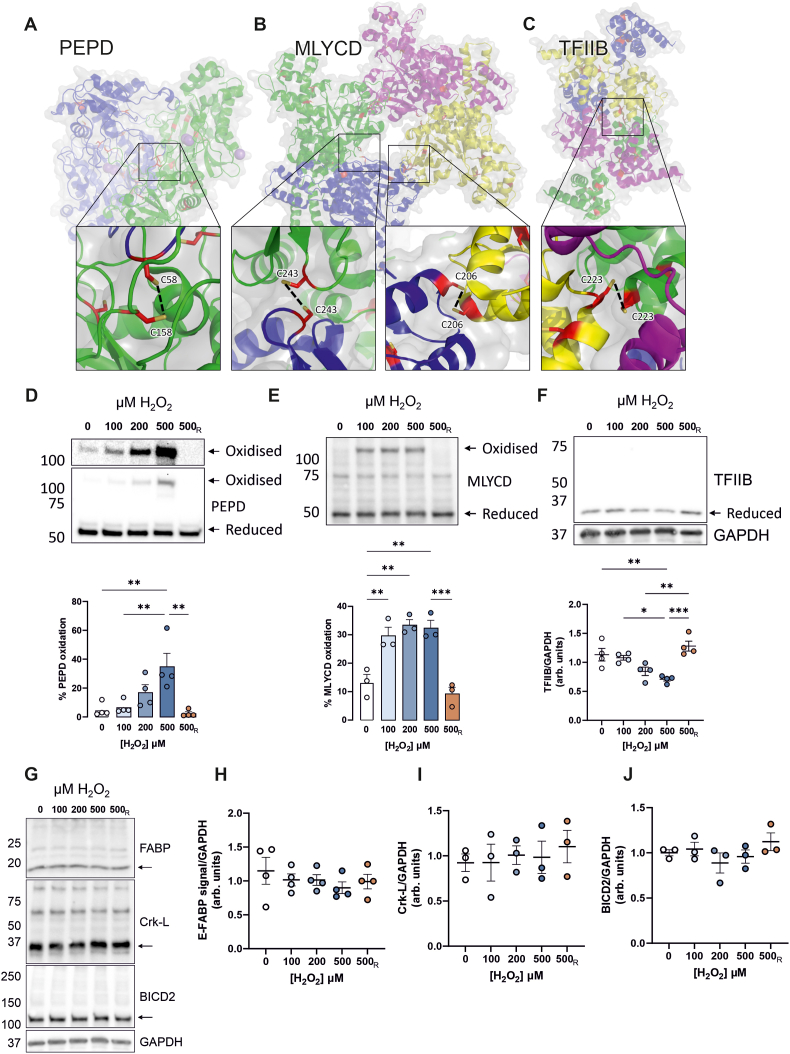
Validation of novel drug-targetable redox-regulated disulphides. (**A**) Crystal structure of PEPD dimer contains a proximal cysteine pair C58–C158 (PDB 2IW2) (**B**) Crystal structure of MLYCD tetramer contains two proximal cysteine pairs C206–C206 and C243–C243 (PDB 4F0X) (**C**) Crystal structure of TFIIB contains a proximal cysteine pair C223–C223 (PDB 5WH1) (**D**) HEK293T treatment with H_2_O_2_ for 15 min causes the formation of a higher MW complex when analysed by non-reducing immunoblot for PEPD, and this signal is reversed with βME (n = 4). (**E**) Treatment with H_2_O_2_ causes higher MW complex formation in immunoblots for MLYCD that is reversed with βME (n = 3). (**F**) Treatment with H_2_O_2_ causes a decrease in TFIIB immunoblot signal that is reversed with βME (n = 4). (**G**) Immunoblots for high-scoring ligandable disulphide candidates that did not show H_2_O_2_-dependent intermolecular disulphide formation, including (**H**) E-FABP (n = 4), (**I**) Crk-L (n = 3), and (**J**) BICD2 (n = 3). (**D-J**) R denotes samples reduced with βME. All data represent mean ± SEM. ∗P < 0.05, ∗∗P < 0.01, ∗∗∗P < 0.001 by 1-way ANOVA with Tukey post hoc test.

We selected the six highest-scoring, ligand-annotated intermolecular cysteine pairs for validation: Xaa-Pro dipeptidase (PEPD), mitochondrial malonyl-CoA decarboxylase (MLYCD), transcription initiation factor IIB (TFIIB), Crk-like protein (Crk-L), protein bicaudal D homolog 2 (BICD2), and fatty acid-binding protein 5 (E-FABP). The structure of PEPD suggests that an intermolecular disulphide could form within the homodimeric protein between C58 and C158 (Fig. 3A). MLYCD has a high-scoring candidate intermolecular disulphide between C206 of two subunits, and a lower-scoring candidate disulphide between C243 of both subunits (Fig. 3B). The structure of TFIIB suggests that an intermolecular disulphide could form between C223 of two TFIIB subunits (Fig. 3C). To determine the sensitivity of these candidates to redox-regulated disulphide formation, HEK293T cells were treated with H_2_O_2_ and analysed by non-reducing immunoblot. When analysed for PEPD, there was a higher molecular weight (MW) moiety that increased in signal in an oxidant-dependent manner (Fig. 3D). This suggests the formation of a redox-regulated intermolecular disulphide bond that was then confirmed through sample treatment with the reducing agent beta-mercaptoethanol (βME), which caused disappearance of the higher MW complex. Similarly, when H_2_O_2_-treated HEK293T cells were analysed for MLYCD, there was an oxidant-dependent increase in a higher MW moiety that was also reducible with βME treatment (Fig. 3E). This is consistent with previous work where purified recombinant MLYCD was found to form an intermolecular disulphide [34]. When H_2_O_2_-treated cells were analysed for TFIIB, there was no detected higher MW complex, but there was an oxidant-dependent decrease in monomer signal (Fig. 3F). This suggests that oxidation of a cysteine in TFIIB is obscuring the antibody epitope or leading to disulphides with several other proteins. Oxidative modification was confirmed by sample treatment with βME, causing the signal to return to levels comparable with the control. Conversely, Crk-L, BICD2, and E-FABP, did not undergo loss in monomer or increase in higher MW complex formation in cells treated with H_2_O_2_, consistent with a lack of redox disulphide formation (Fig. 3G–J).

### 3.4 PEPD contains a redox-regulated intermolecular disulphide bond that modulates p53 activation

**Fig. 4:**
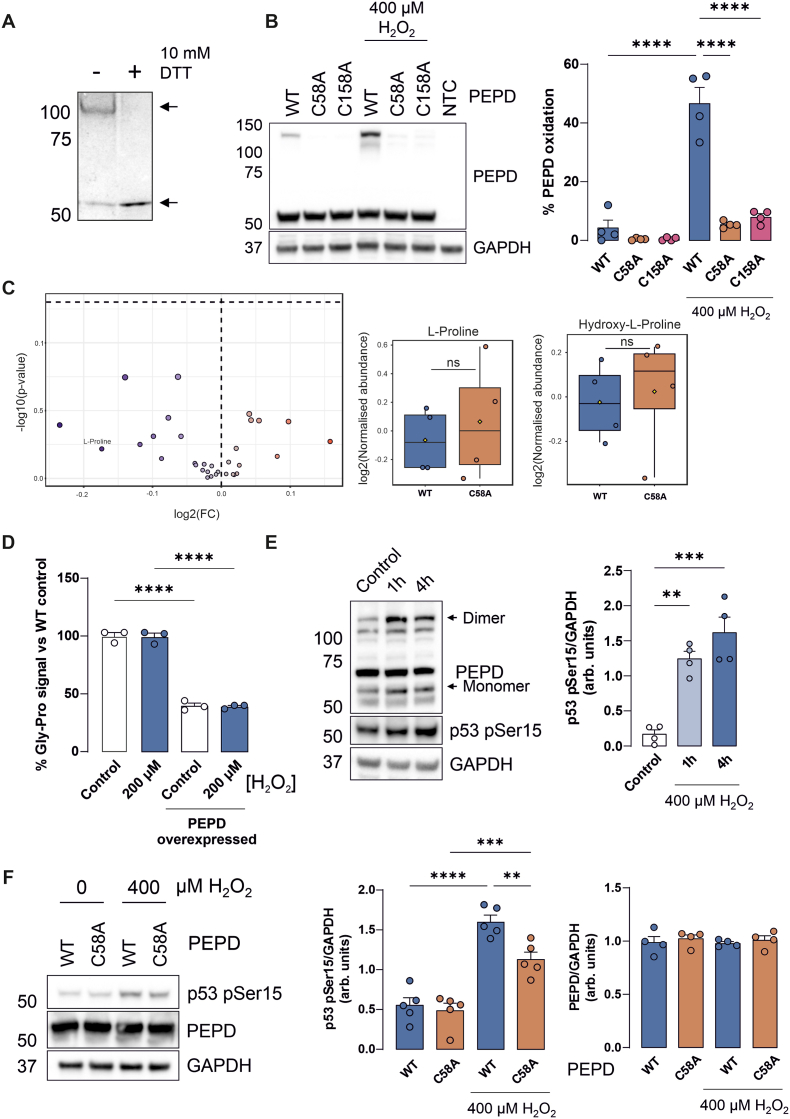
Redox-dependent disulphide formation in PEPD promotes p53 activation. (**A**) Recombinant PEPD forms a disulphide-bound homodimer that can be reduced by treatment with 10 mM DTT. (**B**) In HEK293T cells, transfected WT PEPD is able to form an intermolecular disulphide when treated with H_2_O_2_ for 15 min, which is lost when C58A or C158A PEPD mutants are expressed (n = 4). (**C**) Metabolomics analysis of cells expressing PEPD WT or C58A (n = 3) (**D**) Relative quantification of supplemented Gly-Pro levels in cell lysates with or without PEPD overexpression and in the presence or absence of H_2_O_2_ (n = 3) (**E**) HT1080 cells treated with 400 μM H_2_O_2_ exhibit PEPD oxidation and p53 activation (n = 4). (**F**) HT1080 cells transfected with WT PEPD undergo increased p53 activation after H_2_O_2_ treatment for 1 h, which is attenuated in cells expressing C58A PEPD (n = 5). The levels of PEPD are comparable between cells expressing WT and C58A PEPD (n = 4). All data represent mean ± SEM. ∗∗P < 0.01, ∗∗∗P < 0.001, ∗∗∗∗P < 0.0001 by 1-way ANOVA with Tukey post hoc test.

The redox sensor in PEPD was selected for further characterisation. Firstly, it was confirmed that PEPD formed a reversible disulphide-bound homodimer using recombinant purified protein (Fig. 4A). We next substantiated that the intermolecular disulphide in PEPD was dependent on C58 and C158, the sites identified by ReDisulphID, as mutation of either residue to alanine (C58A or C158A) prevented higher MW complex formation in transfected HEK293T cells treated with H_2_O_2_ (Fig. 4B). To assess the functional impact of intermolecular disulphide formation we initially assessed the effect this may have on the canonical activity of PEPD. Given its role in regulating the intracellular pool of proline through the cleavage of dipeptides with a prolyl or hydroxyprolyl residue in the C-terminal position [35], we measured the abundance of this metabolite. Here we observed no difference in the abundance of proline or any other metabolites in cells expressing WT or C58A PEPD, suggesting that disulphide formation is unlikely to impact on the canonical activity of this enzyme (Fig. 4C). This was further substantiated by directly measuring PEPD activity [31]. In cell lysates, the glycine-proline (Gly-Pro) dipeptide levels after supplementation were decreased in those where PEPD was overexpressed, however this effect was unchanged when PEPD was oxidised (Fig. 4D–S4A). As these findings suggest that disulphide formation does not affect the canonical function of PEPD, we investigated other processes that may be impacted. Given that PEPD also has a non-canonical function in which its interaction suppresses p53 activity, and that this suppression is relieved by H_2_O_2_ [36], we hypothesised that intermolecular disulphide formation in PEPD may mediate this effect. To investigate this we used HT1080 cells as these have functional p53 [37]. Consistent with previous work, treatment of HT1080 cells with H_2_O_2_ induced p53 activation as assessed by measuring phosphorylation of p53 at serine 15 (pSer15) [36], and also enhancing PEPD oxidation (Fig. 4E). To establish if the redox disulphide in PEPD could be responsible for this redox-dependent increase in p53 activity, cells were transfected with WT or C58A PEPD prior to treatment with H_2_O_2_. Notably, treatment with H_2_O_2_ caused increased p53 pSer15 induction in cells expressing WT PEPD compared to those transfected with the C58A mutant (Fig. 4F), indicating that formation of the redox-regulated disulphide in PEPD is involved in the redox-dependent activation of p53.

### 3.5 CL33 activates p53 through covalent modification of C58 in PEPD

**Fig. 5:**
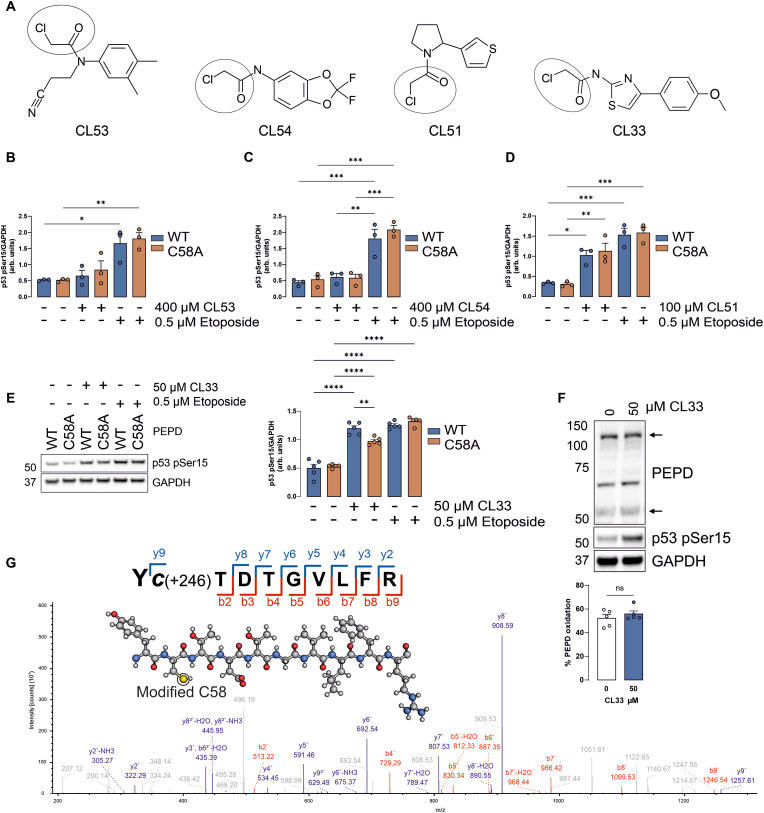
P53 activation through covalent modification of PEPD C58. (**A**) Chemical structures of four compounds previously detected as able to modify PEPD C58 in cells, with their chloroacetamide warheads highlighted. p53 activation was not induced by 1 h treatment of HT1080 cells with (**B**) CL53 or, (**C**) CL54 at 400 μM quantified by immunoblot (n = 3). (**D**) Increased p53 phosphorylation was observed with treatment of HT1080 cells with 100 μM of compound CL51, however this did not differ between cells expressing WT or C58A PEPD (n = 3). (**E**) Treatment of HT1080 with 50 μM CL33 for 1 h increased p53 activation in cells expressing WT PEPD, but significantly less in those expressing C58A PEPD (n = 5). (**F**) Treatment of HT1080 cells with CL33 did not induce disulphide formation of PEPD (n = 4). (**G**) Mass spectrometry identifies a CL33 adduct on C58 in recombinant PEPD. ∗P < 0.05, ∗∗P < 0.01, ∗∗∗P < 0.001, ∗∗∗∗P < 0.0001 by 1-way ANOVA with Tukey post hoc test.

By mapping available chemoproteomic ligandability data to redox sensors in ReDisulphID, several compounds were identified as able to modify C58 of PEPD in treated cells (Fig. 5A) [28], whereas no compounds were identified that modify C158. Given the functional role of the disulphide in PEPD, it was hypothesised that these compounds could enhance p53 activity through adduction of C58 by mimicking the conformational effects of disulphide formation. To test this hypothesis HT1080 cells stably expressing either WT or C58A PEPD were treated with each compound or etoposide as a positive control that induces p53 activation. Here CL53 and CL54 were found not to induce p53 pSer15 (Fig. 5B, C, S4B, C). Whereas in cells treated with CL51, there was a robust induction of p53 pSer15, but this was independent of the redox-regulated disulphide in PEPD, as no differences were observed between cells expressing WT or C58A mutant protein (Fig. 5D–S4D). In contrast, CL33 induced significantly more p53 pSer15 in PEPD WT cells compared to those expressing redox-dead C58A (Fig. 5E), indicating that CL33 mediates p53 activation through modification of C58 in PEPD. Consistent with direct adduction of C58, CL33 did not induce PEPD intermolecular disulphide formation despite activating p53 (Fig. 5F). Moreover, CL33 treatment inhibited PEPD disulphide formation induced by H_2_O_2_ or diamide in HT1080 cells (Fig. S4E and F). This is consistent with direct modification of C58 by CL33, which was substantiated by mass spectrometry analysis of recombinant PEPD treated with this compound (Fig. 5G).

## 4 Discussion

Redox-regulated disulphides serve as crucial redox sensors that mediate key physiological and pathological processes. The thiols involved in these modifications can also be directly targeted, making them valuable therapeutic candidates, yet few have been discovered. Therefore, our aim was to establish an unbiased platform to enable the identification of these redox sensors, as well as compounds that could modify their cysteines. ReDisulphID leverages available structural and chemoproteomic data to reveal novel druggable redox-regulated disulphides. Here we demonstrate how this novel resource can be used to discover and enable targeting of new redox switches by showcasing its utilisation in identifying a redox switch in PEPD that modulates p53 activation, and subsequent discovery of the small molecule CL33 that can target this mechanism.

Several prior algorithms predict whether a cysteine pair forms a disulphide bond. Tools such as DiANNA, DISULFIND, and GDAP accept amino acid sequence as input and use sequence alignment to identify similar structures, inferring disulphide formation based on the proximity of thiol pairs [[38], [39], [40], [41]]. Cysteine Oxidation Prediction Algorithm (COPA) [42], and its successor, the Hb inclusive Algorithm for Cysteine prediction (HAL-Cy) [43], accommodate structural data to predict reactive thiols, including those likely to form disulphides. Additionally, RevssPred compares candidate disulphide bonds to known and reversible disulphides, assessing their likelihood or reversibility based on hydrophobicity and net charge of flanking residues [44]. These tools have proved valuable but lack the ability to systematically discover novel redox-regulated disulphides. We demonstrate, by mining over 1.4 million cysteine pairs and comparing positive and negative targets of reversible disulphide formation, that a small set of geometric and physicochemical descriptors, such as solvent accessibility, pK_a_, and the novel utilisation of crystallographic B-factor, can effectively predict these bonds. Our platform, ReDisulphID, requires no structure or sequence input and allows searches by protein or function. It enriches all solved structures with precomputed solvent accessibility and pK_a_ values for every proximal cysteine pair and enables immediate visualisation of disulphides in their structural context through a user-friendly interface. ReDisulphID identifies thousands of cysteine pairs with high redox scores, frequently found in transcription factors, DNA-repair proteins, regulators of apoptosis, and proteins with numerous other functions. This highlights the widespread role of redox switches in cellular regulation. Furthermore, by overlaying this dataset with chemoproteomic ligandability data, ReDisulphID pinpoints functionally important cysteines amenable to covalent targeting, offering a powerful resource for drug-target discovery. Overlaying redox score with chemoproteomic ligandability data also revealed that predicted redox-regulated disulphides, and particularly cysteines constrained by disulphides in crystal state, were more sparingly labelled by electrophiles in chemoproteomic screens, whereas less proximal cysteines were more frequently ligandable. This suggests that covalent screening datasets poorly represent cysteines involved in regulatory disulphides, likely due in part to limitations in current methodology where disulphide-bound cysteines may transiently mask changes in ligand binding. This reflects previous observations that cellular redox state influences cysteine ligandability [10]. Disulphide bond formation may transiently limit ligand binding, but these cysteines will remain druggable in a redox-dependent manner.

Using ReDisulphID, we identified two novel redox sensors in PEPD and TFIIB and confirmed a previously proposed disulphide in MLYCD. Although an earlier study indicated an intermolecular disulphide in MLYCD that enhanced its activity, this was under artificial in vitro conditions using recombinant protein [34], whereas our study confirms this modification is redox-regulated within cells and highlights that it can be targeted with defined covalent compounds. For example, inhibiting MLYCD is likely to be beneficial in the treatment of heart failure [[45], [46], [47]]. In TFIIB, a redox-regulated disulphide may form between C223 residues in each monomer, however, confirming this modification requires further experimental validation. The ligandability of this cysteine means it may be a potential drug target for regulating TFIIB activity, which could be beneficial in the treatment of cancer, cardiac hypertrophy, or viral pathogenesis [[48], [49], [50]].

Formation of an intermolecular disulphide between C58 and C158 residues within the PEPD dimer is consistent with this cysteine pair having a close proximity and a low calculated pK_a_ of ∼5, thus substantially deprotonated and susceptible to oxidation at physiological pH. The PEPD disulphide was detected under basal conditions and was further induced by 200 μM extracellular H_2_O_2_, corresponding to an estimated intracellular concentration of ∼300 nM when accounting for an approximately 650-fold transmembrane gradient [51]. This concentration remains within the physiologically relevant range [52], and is particularly relevant when localised redox microenvironments are considered. Due to the likely functional importance of this disulphide it was hypothesised that it may impact on the canonical function of PEPD in hydrolysing dipeptides with C-terminal prolines or its non-canonical role in interacting and suppressing p53 activity [36]. By characterising the redox-regulated disulphide in PEPD it was found that formation of the disulphide between C58 and C158 enhanced p53 activation without perturbing canonical prolidase activity. We tested four compounds that were previously found to modify cysteine C58 [28], thus having potential to also modulate the PEPD-p53 axis. CL53 or CL54 did not induce p53 phosphorylation. This may reflect an inability of these compounds to modify PEPD C58 in HT1080 cells, or that the modification they induce is insufficient to recapitulate the effect of disulphide bond formation. Whereas CL51 caused p53 activation that was independent of C58. It is conceivable that CL51 instead activates p53 through modification of C158 on PEPD. However, as chemoproteomic studies that detected CL51-dependent modification of C58 did not observe corresponding modification of C158, it is more likely that this compound acts through PEPD-independent pathways that regulate p53. Finally, compound CL33 enhanced p53 phosphorylation in a PEPD C58-dependent manner. CL33 contains a chloroacetamide warhead that can alkylate C58, which may disrupt binding between PEPD and p53 by mimicking the conformational effect of disulphide formation. Disrupting the PEPD-p53 interaction has been found to restore transcriptional activity to several oncogenic p53 mutants [53], thus CL33 could be further developed for targeting tumours that express mutated p53.

Our work establishes ReDisulphID, a new platform for identifying redox sensors and linking them to drug discovery. Using this resource, we uncovered novel druggable redox sensors, including a sensor in PEPD and a compound that modulates p53 via this site. These findings highlight the translational potential of ReDisulphID and provide a foundation for the systematic discovery of druggable modulators of redox signalling.

### 4.1 Statistical analysis

Differences between groups were assessed using ANOVA followed by a two-tailed unpaired Student's t-test, or for multiple comparisons a Tukey's test. Data are shown as mean ± SEM and results were considered significant at a 5% significance level.

## CRediT authorship contribution statement

**Pierre Coleman:** Conceptualization, Investigation, Methodology, Software, Writing – original draft, Writing – review & editing. **Anna Laddach:** Investigation, Methodology. **Rhys Anderson:** Methodology, Resources. **Xiaoping Yang:** Investigation, Methodology. **Ravi Kumar:** Investigation, Methodology. **Ajay Shah:** Methodology, Supervision. **Franca Fraternali:** Methodology, Supervision. **Joseph R. Burgoyne:** Conceptualization, Funding acquisition, Methodology, Supervision, Writing – review & editing.

## Declaration of competing interest

We confirm the authors have no conflict of interest to declare.

## Data Availability

ReDisulphID is available at redisulphid.net. Code can be found at github.com/piecole/strucseq. All other data are available in the main text or the supplementary materials.
