# Supplementary material for: ReDisulphID: A discovery platform for thiol redox sensors identifies a druggable site regulating p53 activation
Source: Redox Biol. 2026 Apr 29;94:104196. doi: 10.1016/j.redox.2026.104196 (PMC13157088; doi:10.1016/j.redox.2026.104196)
Supplement: Multimedia component 1 [file mmc1.docx]

**Supplemental Material**

**
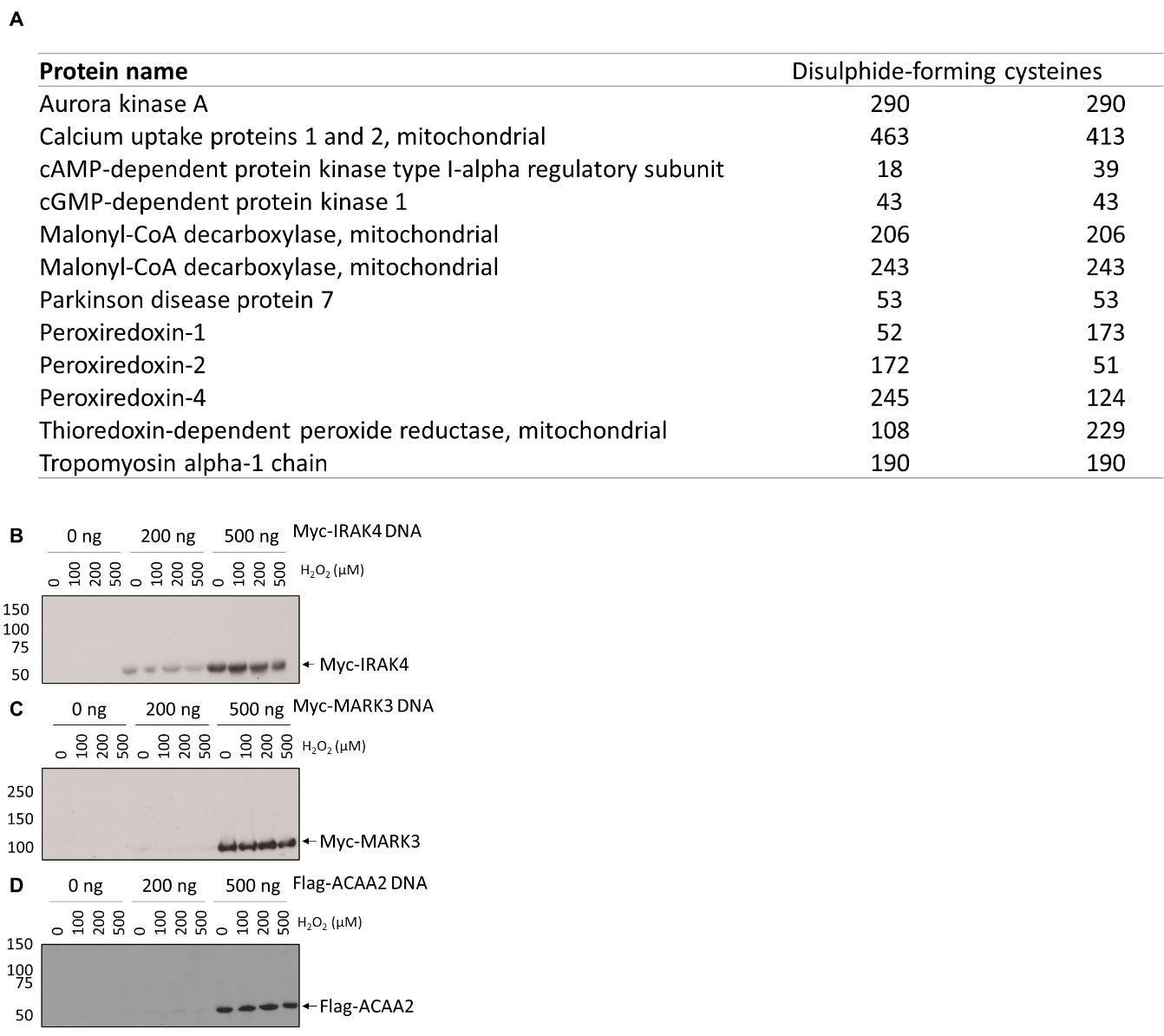
**

**Fig. S1**

(**A**) Known intermolecular redox-regulated disulphides with high-quality protein structures. (**B-D**) HEK293T cells were transfected with plasmids for (**B**) Myc-IRAK4 (**C**) Myc-MARK3 (**D**) FLAG-ACAA2 for 24 hours then treated with or without H_2_O_2_ for 15 min followed by non-reducing immunoblot analysis.

**
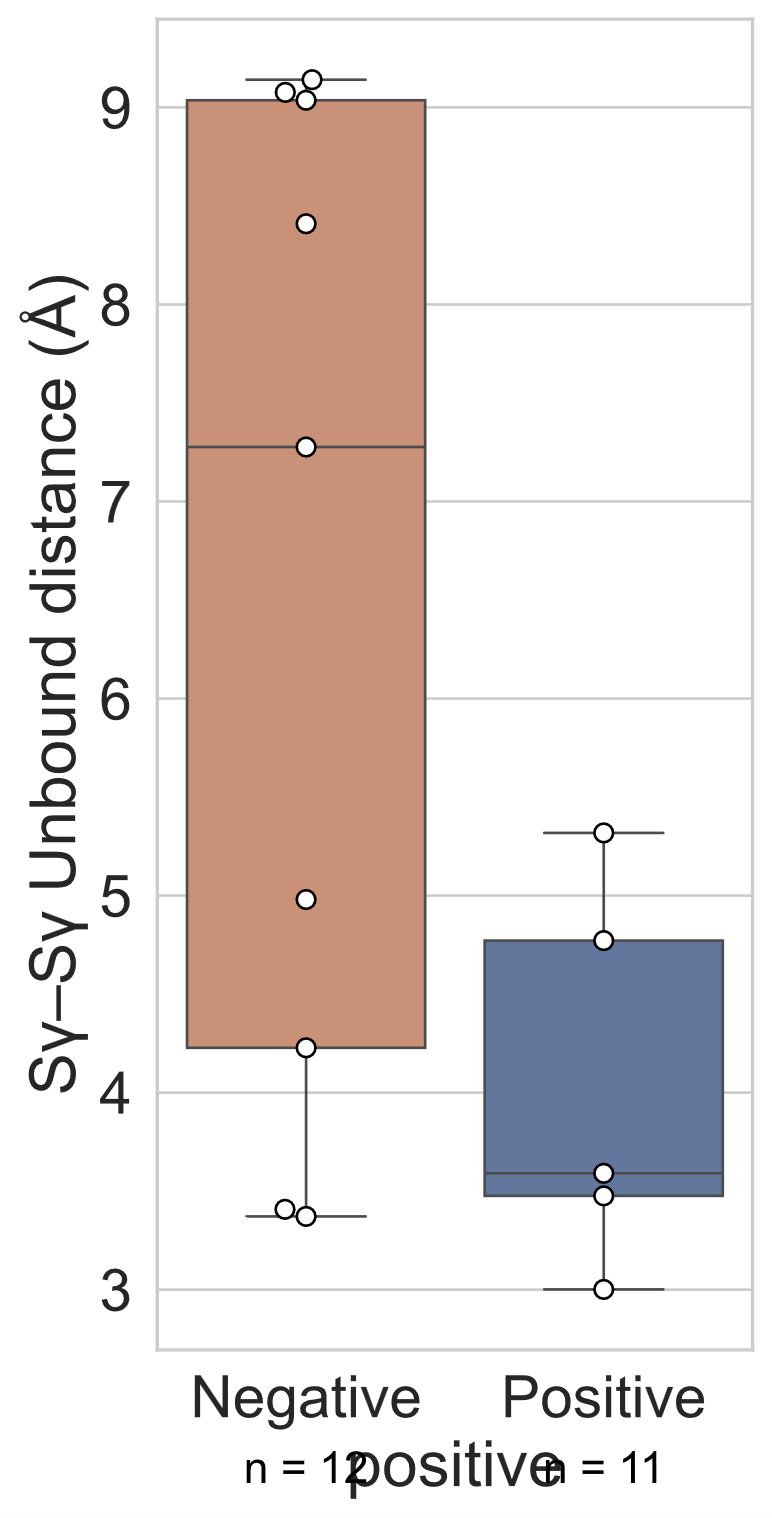
**

**Fig. S2**

Thiol separation between positive and negative candidate disulphides remains different with disulphide-bound structures omitted. *P < 0.05 statistical significance (Welch’s t-test two tailed), n = 9 for negative candidates, n = 5 for positive candidates.

**
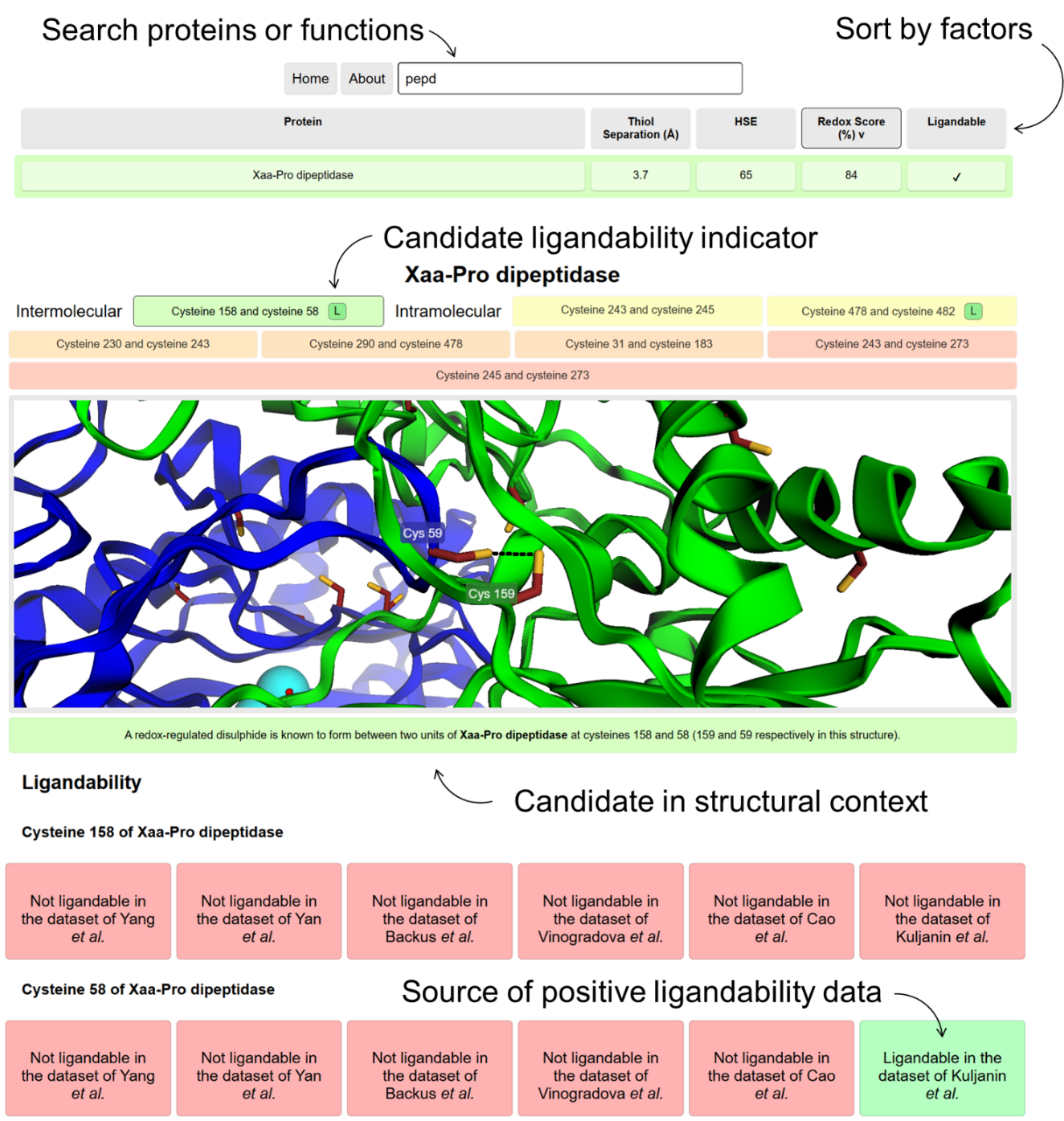
**

**Fig. S3**

ReDisulphID is a user-friendly resource for browsing ligandable disulphides predicted from structural data.


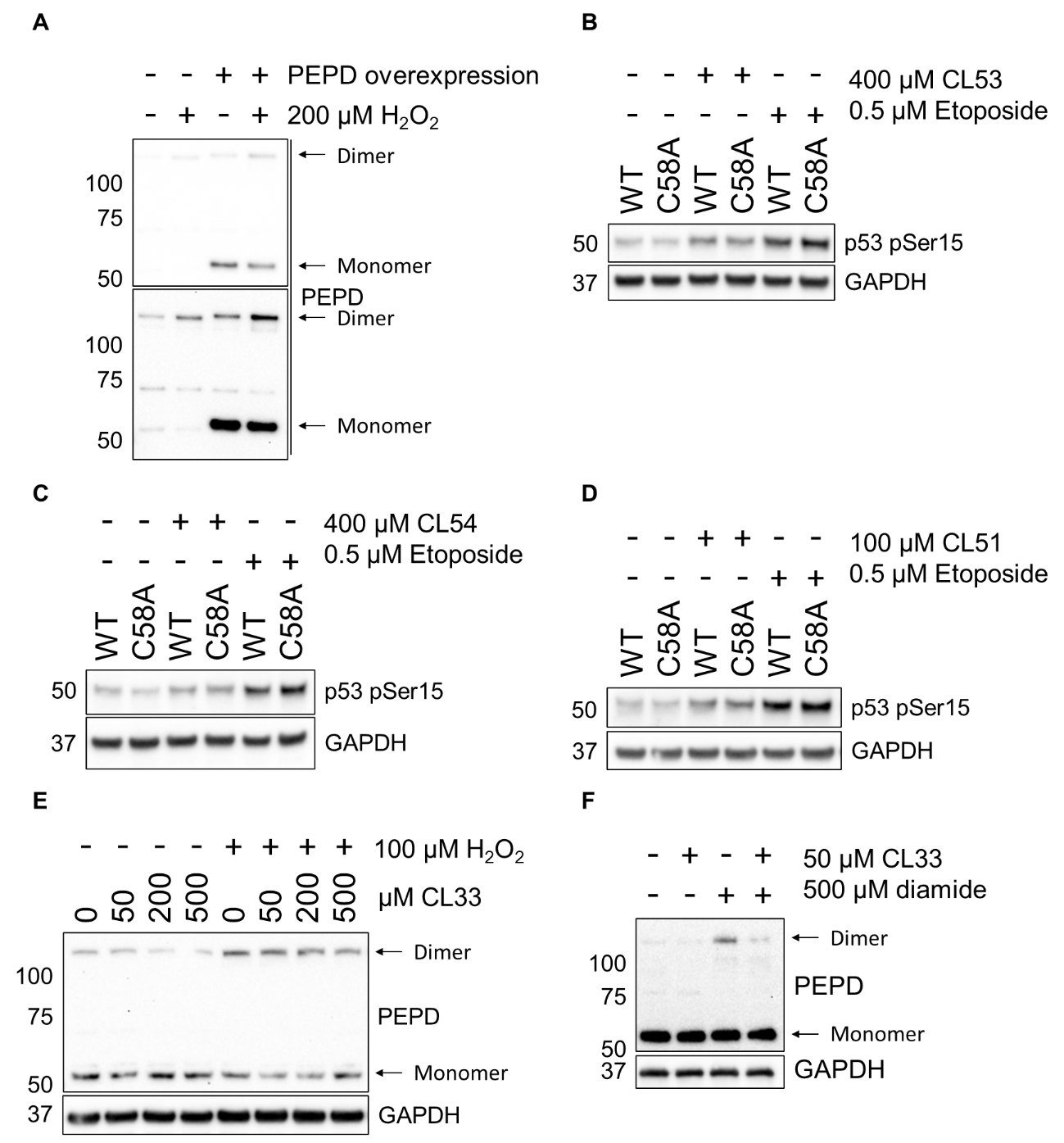


**Fig. S4**

(**A**) Immunoblot analysis for oxidation of PEPD in cell lysates with or without PEPD overexpression or H_2_O_2_ in samples used for gly-pro quantification. (**B-D**) Representative immunoblot analysis of p53 phosphorylation in HT1080 cells expressing WT or C58A PEPD treated with (**B**) 400 μM CL53, (**C**) 400 μM CL54, or (**D**) 100 μM CL51. (**E**) Immunoblot analysis of HT1080 cells treated with CL33 for 1 hour and then H_2_O_2_ for 15 minutes. (**F**) Immunoblot analysis HT1080 cells treated with CL33 and diamide for 15 minutes.
